# Comparing the Gut Microbiome in Autism and Preclinical Models: A Systematic Review

**DOI:** 10.3389/fcimb.2022.905841

**Published:** 2022-07-01

**Authors:** Mohammed U. Alamoudi, Suzanne Hosie, Anya E. Shindler, Jennifer L. Wood, Ashley E. Franks, Elisa L. Hill-Yardin

**Affiliations:** ^1^ School of Health and Biomedical Sciences, Royal Melbourne Institute of Technology (RMIT), Bundoora, VIC, Australia; ^2^ Medical Laboratory Technology Department, Faculty of Applied Medical Sciences, Jazan University, Jazan, Saudi Arabia; ^3^ Department of Microbiology, Anatomy, Physiology and Pharmacology, School of Life Sciences, La Trobe University, Bundoora, VIC, Australia

**Keywords:** autism, mouse model, gastrointestinal tract, microbiota, genetics, environmental

## Abstract

Many individuals diagnosed with autism spectrum disorder (ASD) experience gastrointestinal (GI) dysfunction and show microbial dysbiosis. Variation in gut microbial populations is associated with increased risk for GI symptoms such as chronic constipation and diarrhoea, which decrease quality of life. Several preclinical models of autism also demonstrate microbial dysbiosis. Given that much pre-clinical research is conducted in mouse models, it is important to understand the similarities and differences between the gut microbiome in humans and these models in the context of autism. We conducted a systematic review of the literature using PubMed, ProQuest and Scopus databases to compare microbiome profiles of patients with autism and transgenic (NL3^R451C^, Shank3 KO, 15q dup), phenotype-first (BTBR) and environmental (Poly I:C, Maternal Inflammation Activation (MIA), valproate) mouse models of autism. Overall, we report changes in fecal microbial communities relevant to ASD based on both clinical and preclinical studies. Here, we identify an overlapping cluster of genera that are modified in both fecal samples from individuals with ASD and mouse models of autism. Specifically, we describe an increased abundance of *Bilophila*, *Clostridium*, *Dorea* and *Lactobacillus* and a decrease in *Blautia* genera in both humans and rodents relevant to this disorder. Studies in both humans and mice highlighted multidirectional changes in abundance (i.e. in some cases increased abundance whereas other reports showed decreases) for several genera including *Akkermansia*, *Bacteroides*, *Bifidobacterium*, *Parabacteroides* and *Prevotella*, suggesting that these genera may be susceptible to modification in autism. Identification of these microbial profiles may assist in characterising underlying biological mechanisms involving host-microbe interactions and provide future therapeutic targets for improving gut health in autism.

## Introduction

Autism spectrum disorder (ASD) is a heterogeneous neurodevelopmental condition diagnosed based on stereotyped or repetitive patterns of behavior, impaired communication interactions as well as language delay (American Psychiatric Association, 2013). Several studies also indicate that changes in the gut microbiome are associated with ASD (Strati et al., 2017). Individuals with ASD commonly experience various comorbidities including intellectual disability, sleeplessness, immune disorders, metabolic impairments, anxiety, epilepsy and gastrointestinal (GI) disorders (Matson et al., 2011; Hsiao, 2014; Lai et al., 2019). An assessment of the prevalence of GI symptoms in autism revealed as many as 70-90% of individuals diagnosed with ASD reported gut dysfunction (Adams et al., 2011; Gorrindo et al., 2012). People with ASD frequently report moderate to severe diarrhea, constipation as well as abdominal pain (Holingue et al., 2018) and increased gut permeability (Esnafoglu et al., 2017). In addition, GI problems experienced by children with ASD tend to be more frequent and severe than those reported in neuro-typical children (Vuong and Hsiao, 2017). In the autistic population, GI symptoms also correlate with the severity of core autism symptom such as sensory issues, sleep problems, comorbid psychopathology, challenging behavior, communication and language difficulties, increased social withdrawal and anxiety levels as well as irritability (Gorrindo et al., 2012; Attlee et al., 2015).

GI dysfunction experienced by individuals with ASD is likely driven by disruption of the microbiota-gut brain axis. This crucial interplay between the gut microbiome, the nervous system and the immune system maintains homeostatic microbial levels and maintains health. Changes in each of these domains are well established in both clinical and preclinical studies of autism (Bourgeron, 2009; Lee et al., 2020), Margolis et al., 2021). Nervous system changes occurring in autism not only affect the brain but may also alter the intrinsic nervous system of the gut (the enteric nervous system; ENS) which is in constant crosstalk with the GI microbiome and the immune system (i.e., reviewed in Margolis et al. (2021)). Dysbiosis; a reduction in microbial diversity leading to an increase in pathogenic bacteria is common in ASD patients (Valicenti-McDermott et al., 2006; Sekirov et al., 2010; Clemente et al., 2012; Martín et al., 2014; Luna et al., 2017; Thursby and Juge, 2017; Almeida et al., 2019; Rinninella et al., 2019; Xu et al., 2019b), but factors such as genetics, diet and living environment also influence the gut microbiome. An ideal approach for assessing changes in the microbiome in the absence of confounding factors is by studying preclinical models of ASD. In line with clinical findings, studies in a range of mouse models of autism also show altered microbial profiles (Hsiao, 2014; Hosie et al., 2019; Sgritta et al., 2019; Sharon et al., 2019).

Despite evidence for dysbiosis in both clinical and mouse model studies further characterisation of microbiome profiles across both clinical and animal model datasets is needed to pinpoint how microbial changes impact gut physiology in people with ASD. Identifying overlapping patterns of microbial variation in clinical populations and mouse models of autism will provide the basis for modulating the microbiome to improve gut health in individuals with ASD. Therefore, the characterization of gut microbial dysbiosis in people with ASD may provide insights into the underlying mechanisms of GI dysfunction symptoms in autism.

### Factors Influencing Microbial Dysbiosis in Autism

The gut microbiome is influenced by multiple interlinked factors including genetics, the environment (stress, diet, lifestyle) and/or immune status. The microbiome interacts directly with both the immune and nervous system to modulate not only gut function but also influence mood and behaviour (Lozupone et al., 2012). Children with ASD frequently show dysbiosis (Finegold et al., 2010; De Angelis et al., 2013; Luna et al., 2017; Strati et al., 2017) and appear to be more susceptible to immune dysfunction and increased intestinal permeability compared to the general population (Strati et al., 2017; Rose et al., 2018; Matta et al., 2019). Characterization of microbial profiles will not only enable the identification of which microbes are altered in autism but may also help to understand the influence of the microbiome in ASD.

Genetic changes are prominent in ASD with more than 1000 single nucleotide (point) mutations associated with this disorder (Levy et al., 2011; Sanders et al., 2011; Guang et al., 2018). As yet, the precise relationship between host genetics and microbial dysbiosis is for the most part unknown. However, a study by Liu et al., for example, provides mechanistic evidence where host genetics shape the fecal microbial community (Liu et al., 2016). Many ASD-associated mutations occur in genes that encode scaffolding proteins, synaptic cytoplasmic and cell-adhesion molecules as well as various molecules involved in neurotransmission or regulation of synaptic protein synthesis (Guang et al., 2018). For example, a missense mutation in the neuroligin 3 (Nlgn3) protein was identified in autism patients (Jamain et al., 2003). Nlgn3 regulates neuronal morphology and dendritic outgrowth (Xu et al., 2019a) and impairs synaptic function in the central nervous system (Tabuchi et al., 2007; Etherton et al., 2011; Hosie et al., 2018). Multiple mutations associated with ASD also occur in the gene encoding the synaptic scaffolding protein ‘SH3 and multiple ankyrin repeat domains 3’ (SHANK3) (Durand et al., 2007). Alterations in SHANK3 modify cortical neuronal connectivity and increase the risk of ASD diagnosis (Pagani et al., 2019). These same mutations modify the enteric nervous system leading to abnormal gastrointestinal motility and structural changes in the GI tract (Hosie et al., 2019; Sauer et al., 2019; Herath et al., 2020; Lee et al., 2020; Leembruggen et al., 2020).

In addition to genetic influences, environmental factors can modify microbial profiles and could contribute to the etiology of complex disorders. Several medications, including the anti-epileptic drug, valproate, modify gut microbial function including fatty acid production (Poolchanuan et al., 2020). It has also been shown that the risk of ASD diagnosis is increased in infants born to mothers prescribed valproate (Christensen et al., 2013). In keeping with clinical findings, maternal immune dysregulation is associated with microbial dysbiosis in rodent offspring (Hsiao et al., 2013) demonstrating the importance of immune-microbial crosstalk during development. It is therefore clinically relevant to assess changes in the microbiome due to both genetic and environmental influences.

### Mouse Models Relevant to ASD

Gut microbial changes and the development of ASD-relevant behaviors have been assessed in multiple preclinical models to assist in unravelling the impacts of autism-associated gene mutations on gut microbiota (Newell et al., 2016; Coretti et al., 2017; Golubeva et al., 2017; Lim et al., 2017; Cristiano et al., 2018; Hosie et al., 2019; Sauer et al., 2019; Sgritta et al., 2019; Septyaningtrias et al., 2020). To understand the mechanisms underlying microbial changes in autism, mouse models are commonly used to highlight shifts in microbial profiles in a controlled environment.

It is important to define microbial profiles in mouse models of ASD to understand how host-microbe interactions might influence GI dysfunction. The NL3^R451C^, SHANK3 KO and 15q dup mice express autism-associated gene mutations/duplications and show autism-relevant behaviors such as repetitive grooming and reduced interest in novel mice (Tabuchi et al., 2007; Nakatani et al., 2009; Peça et al., 2011). Both NL3^R451C^ and SHANK3 KO mice demonstrate GI dysfunction (Hosie et al., 2019; Sauer et al., 2019). NL3^R451C^ mice show altered faecal microbial populations (Hosie et al., 2019) and SHANK3 KO mice also exhibit shifts in the microbiome (Sauer et al., 2019); Sgritta et al., 2019). In addition to transgenic models, phenotype-first models such as Black and Tan BRachyury (BTBR) mice show behavioral endophenotypes relevant to ASD symptoms, including increased repetitive self-grooming behaviors, abnormal ultrasonic vocalization patterns and well as social approach and reciprocal social interaction deficits (McFarlane et al., 2008; Scattoni et al., 2008; Pearson et al., 2011; Pobbe et al., 2011).

Here we analyzed microbial data from six well-established mouse models of ASD that resulted from our systematic search terms. In addition to highlighting similarities across these datasets, we compare mouse model and clinical ASD microbial studies. Given that shifts in microbial profiles frequently correlate with GI symptoms, a better understanding of how microbes impact the host will enhance treatments for GI dysfunction in patients with ASD. Here we have undertaken a systematic review of the literature to compare preclinical and clinical studies of the microbiome in the context of autism spectrum disorder.

## Materials and Methods

An electronic literature search was conducted in PubMed, ProQuest and Scopus database engines to identify appropriate studies using the following medical subject heading terms and text words: “Autism” AND “Gastrointestinal” AND “Microbe OR Microbiome OR Microbiota”. Selected studies were limited to English language studies and there was no restriction regarding age groups, gender/sex, time frame or location. The Preferred Reporting Items for Systematic Reviews and Meta-Analyses (PRISMA) guidelines were applied for the included studies (Page et al., 2021).

### Selection Criteria for Study Inclusion/Exclusion

The criteria set for this systematic review included inclusion/exclusion criteria and study eligibility. The inclusion criteria were as follows: primary studies focused only on data relating to changes in the GI microbiome sampled from people with autism and from mouse models of autism. There were no limitations regarding age groups, gender/sex, time frame or location. Exclusion criteria applied included: discarding any study focused on species other than human or mice, studies that did not assess microbial diversity (such as studies focused on specific microbes), studies that did not use molecular methods for microbial analysis, or that included subjects receiving specific treatments or factors which influence the GI microbial community. Furthermore, studies that did not provide relevant results but instead focused on the GI microbiome products such as chemical metabolites or toxins, studies that were not primary articles such as reviews, case reports or meta-analysis studies were omitted.

**Figure 1:**
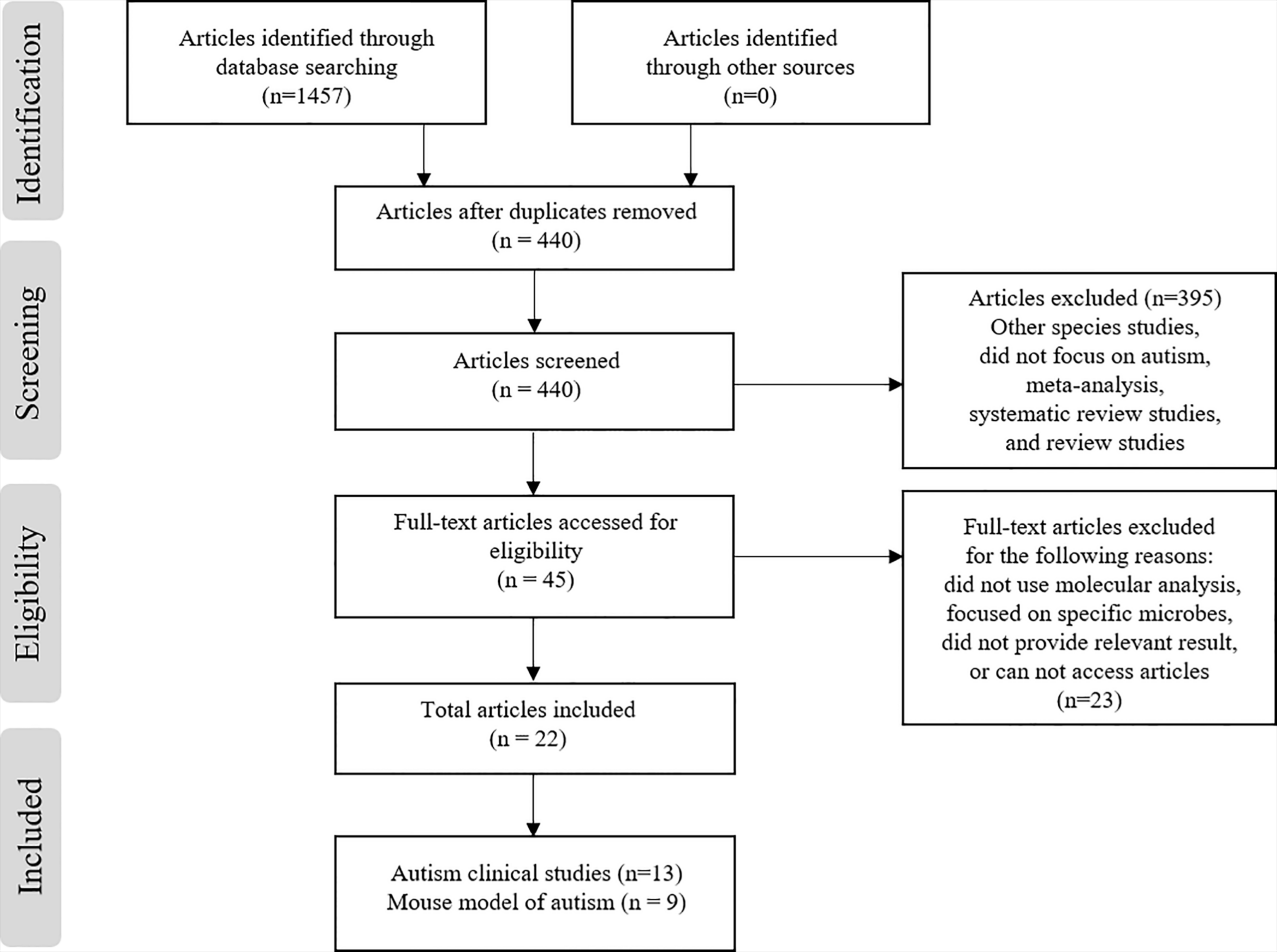
Study screening and selection process.

The PubMed, ProQuest, and Scopus electronic database searches identified 1457 articles (n = 281, 499, and 677, respectively) based on screening the titles and abstracts for the search terms used (**Figure 1**). From these articles, 21 were selected (13 clinical studies and 9 articles detailing ASD mouse models). During the screening process, a total of 1412 articles out of 1457 were removed due to duplication (n= 1017), other species studies such as rodent or hamster studies (n= 5), containing meta-analysis or being a systematic review (n= 22), or were narrative reviews or non-English studies (n= 373). Afterwards, the remaining 45 articles were screened for eligibility (full-text assessment), 22 articles were fully assessed for eligibility and 23 articles were excluded for the following reasons; the study method did not use molecular analysis for microbial diversity (n= 3), the study focused on specific microbes or species only (n= 9), the study entailed the use of factors that influence the microbiome such as drug treatment or probiotic supplementation (n= 6), or the study did not provide relevant results or specify ASD (n= 5). Finally, 22 studies met the inclusion criteria and were extracted for analysis. Among the 22 studies, 13 reported clinical cases of ASD patients (**Table 1**) and 9 reported microbial diversity within mouse models of ASD (3 studies for mouse models using environmental factors and 6 studies in genetic mouse models) (**Table 2**).

**Table 1:** Quality assessment for each clinical study.

Item		References (n=13)												
		Berding & Donovan (2020)	Carissimi et al. (2019)	Dan et al. (2020)	Gondalia et al. (2012)	Kong et al. (2019)	Liu et al. (2019)	Niu et al. (2019)	Rose et al. (2018)	Son et al. (2015)	Strati et al. (2017)	Wang et al. (2020)	Zou et al. (2020)	Zurita et al. (2020)
1	Research question or objective clearly stated	2	2	2	2	2	2	2	2	2	2	2	2	2
2	Appropriate sample size	2	2	2	2	2	2	2	2	2	2	2	2	2
3	Study population clearly specified and defined	2	2	2	2	2	2	2	2	2	2	2	2	2
4	Sample recruited from the same or similar populations	2	2	2	2	2	2	2	2	2	2	2	2	2
5	Sample recruited at the same time period	0	0	0	0	0	0	0	0	0	0	0	0	0
6	Inclusion and exclusion criteria	2	1	2	2	2	2	2	2	1	1	2	2	1
7	Outcome measures clearly defined, valid, reliable, and implemented consistently across all study participants	2	2	2	2	2	2	2	2	2	2	2	2	2
8	Blinding of participants and personnel	0	0	0	0	0	0	0	0	0	0	0	0	0
9	Adequate statistical analyses	2	2	2	2	2	2	2	2	2	2	2	2	2
10	Other bias measured and adjusted statistically for their impact on the outcomes	0	0	0	0	0	0	0	0	0	0	0	0	0
Risk of bias score		14	13	14	14	14	14	14	14	13	13	14	14	13

**Table 2:** Quality assessment for each mouse model study.

Item		References (n=9)								
		Hosie et al. (2019)	Cristiano et al. (2018)	Golubeva et al. (2017)	Hsiao et al. (2013)	Lim et al. (2017)	Newell et al. (2016)	Sauer et al. (2019)	Septyaningtrias et al. (2020)	Sharon et al. (2019)
1	Research question or objective clearly stated	2	2	2	2	2	2	2	2	2
2	Appropriate sample size	2	1	1	2	2	2	1	1	2
3	Study population clearly specified and defined	2	2	2	2	2	2	2	2	2
4	Sample recruited from the same or similar populations	2	2	2	2	2	2	2	2	2
5	Sample recruited at the same time period	2	2	2	2	2	2	0	2	2
6	Inclusion and exclusion criteria	0	0	0	0	0	0	0	0	0
7	Outcome measures clearly defined, valid, reliable, and implemented consistently across all study participants	2	2	2	2	2	2	2	2	2
8	Blinding of participants and personnel	0	0	0	0	0	0	0	0	0
9	Adequate statistical analyses	2	2	2	2	2	2	2	2	2
10	Other bias measured and adjusted statistically for their impact on the outcomes	0	0	0	0	0	0	0	0	0
Risk of bias score		14	13	13	14	14	14	11	13	14

### Data Extraction

A data extraction form was designed for the eligible studies based on the selection criteria applied [61]. The information extracted for analysis included: the first author’s name, year of publication, species (human or mice), sample size, gender for human and sex for mice, background strain (mice only), type of mutation or environmental agent (mice only), type of specimen collected for analysis, diagnostic methods used for microbial diversity, the unit used to describe microbial community results, microbial taxonomic rank (i.e., phylum, order, class or family).

## Results

### Predominant Properties of the GI Microbiome in ASD

Thirteen clinical studies met the selection criteria and were assessed in the current study. Most cases reported were male children, where the percentage of males exceeded 80% of the total cases in each study. DNA extraction from faecal specimens and next generation sequencing 16S rRNA gene sequencing were the main methods used for all studies. Since bacteria within gut microbial populations are grouped/classified taxonomically into species, genera, families, orders and classes as well as phyla, analytical methods of interest for this review appropriately classified gut bacterial groupings. Specifically, all studies included in the current review classified microbial taxa and diversity using operational taxonomic unit (OTU) calling using 97% similarity thresholds.

Gut microbial differences occurring in individuals with ASD were compared (**Table 3**). Overall, in both ASD individuals and control individuals Firmicutes and Bacteroidetes were the most predominant microbial taxa while Proteobacteria and Actinobacteria were the least predominant phyla (Son et al., 2015; Strati et al., 2017; Carissimi et al., 2019; Kong et al., 2019; Liu et al., 2019; Niu et al., 2019; Dan et al., 2020; Zou et al., 2020). In individuals with ASD, four studies reported an increase in the Firmicutes/Bacteroidetes ratio driven by either a decrease in Bacteroidetes (Strati et al., 2017; Kong et al., 2019; Niu et al., 2019) or an increase in Firmicutes (Dan et al., 2020). Conversely, two studies reported a decrease in the Firmicutes/Bacteroidetes ratio in ASD individuals driven by a reduction in Firmicutes (Liu et al., 2019; Zou et al., 2020).

**Table 3:** Properties of the GI microbiome from faecal samples in ASD clinical cases.

	Publication	Sample size	Method	Bacterial changes in ASD individuals compared to neurotypical controls at different classification levels
**1**	Berding & Donovan (2020)	26 ASD and 32 control children (2-7 years)	16S rRNA gene sequencing of V3 -V4 region using Illumina MiSeq platform	ASD individuals showed variation in their microbial taxa over time. Taxa identified to show significant variation were Clostridiaceae, Streptophyta, and Clostridiaceae Clostridium.
**2**	Carissimi et al. (2019)	16 ASD males and 7 (2 male, 5 female) control children (2-6 years)	Shotgun Metagenomics analysis, Paired End (PE) sequencing	Reduced microbial richness in ASD individuals compared to controls as shown by a lower alpha diversity (Fisher index analysis) in ASD individuals
**3**	Dan et al. (2020)	143 ASD (127 male, 16 female) and 143 (130 male, 13 female) control children (1-13 years)	16S rRNA gene sequencing of V4 region Illumina Hiseq X platform	Phylum level: higher Firmicutes levels and a significantly higher Firmicutes/Bacteroidetes ratio (higher Proteobacteria and Actinobacteria.). Genus level: *Bacteroidetes* were significantly decreased while *Dialister*, *Escherichia-Shigella* and *Bifidobacterium* were increased
**4**	Gondalia et al. (2012)	51 ASD (42 male, 9 female) and 53 (19 male, 34 female) neurotypical sibling controls (2-12 years)	16S bacterial sequences of full amplicon using massively parallel bacterial tag-encoded FLX 16s rDNA amplicon pyrosequencing (bTEFAP). Roche 454 FLX platform.	Phylum level: No significant difference Genus level: increased *Catenibacterium*
**5**	Kong et al. (2019)	20 ASD (15 male, 5 female) and 19 (8 male, 11 female) family members (7-25 years)	16S rRNA gene sequencing of V3 -V4 region using Illumina MiSeq platform	Phylum level: No difference in overall abundance however identified a higher Firmicutes/Bacteroidetes ratio in ASD individuals driven by a reduction of Bacteroidetes. Higher chance of Proteobacteria overgrowth in ASD. Genus level: showed trends of altered abundance between ASD and control in *Paraprevotella, Granulicatella, Butyricimonas, cc_115, Peptoniphilu*s and *Eubacterium*
**6**	Liu et al. (2019)	30 ASD and 20 neurotypical controls (2.4-18 years)	16S rRNA gene sequencing of the V3-V4 region using Illumina MiSeq platform	Phylum level: Less species diversity in ASD. Firmicutes was decreased and Acidobacteria was increased. Family level: *Veillonellaceae*, *Pseudomonadaceae* and *Enterobacteriaceae* were enriched, whereas *Ruminococcaceae, Streptococcaceae, Peptostreptococcaceae* and *Erysipelotrichaceae* were significantly decreased Genus level: increase in *Megamonas*.
**7**	Niu et al. (2019)	114 ASD and 40 neurotypical control children (3-8 years)	16S rRNA gene sequencing of the V3-V4 using Illumina MiSeq platform	Phylum level: reduced Bacteroidetes and Actinobacteria and increased Proteobacteria and Firmicutes creating an increased Firmicutes/Bacteroidetes ratio. Genus level: lower abundance of *Bacteroides*, *Bifidobacterium*, *Ruminococcus*, *Roseburia* and *Blautia*.
**8**	Rose et al. (2018)	50 ASD (42 males, 8 Females) and 41 (38 males, 3 females) neurotypical controls. (3-12 years)	16S rRNA gene sequencing of V3-V4 regions with Illumina MiSeq platform	Phylum level: No significant difference Family level: increased relative abundance of *Bacteriodaceae, Lachnospiraceae, Prevotellaceae* and *Ruminococcaceae* in individuals with ASD and gut symptoms
**9**	Son et al. (2015)	59 ASD (52 male, 8 Female) and 44 neurotypical siblings (7-14 years}	16S rRNA gene sequencing of V1-V2 and V1-V3 regions using Illumina MiSeq platform	Phylum level: No significant difference
**10**	Strati et al. (2017)	40 ASD (31 male, 9 female) and 40 (28 male, 12 female) neurotypical children	Bacterial 16S rRNA gene sequencing of V3–V5 regions and internal transcribed spacer of fungal ITS1 rDNA	Phylum level: increased Firmicutes/Bacteroidetes ratio due to reduction of Bacteroidete*s*. Genus level: increase in *Collinsella*, *Corynebacterium*, *Dorea* and *Lactobacillus* and decrease of *Alistipes, Bilophila, Dialister, Parabacteroides*, and *Veillonella* Mycological genus level: No difference in *Aspergillus, Candida, Penicillium* and *Malassezia*
**11**	Wang et al. (2020)	26 ASD (24 male, 2 female) and 24 (22 males, 2 females) neurotypical children (2-8 years)	16S rRNA gene sequencing of V1-V2 region using Illumina HiSeq platform	Phylum level: no difference in Firmicutes/Bacteroidetes ratio but found a significant decrease in the abundance of Actinobacteria. Family Level: increase in *Rikenellaceae* and decrease in *Veillonellaceae* and *Bifidobacteriaceae* Genus level: *Bifidobacterium* were decreased, while abundance of *Clostridium*, *Oscillospira*, *Ruminococcus*, *Odoribacter, Cetobacterium*, and *Victivallales* were increased. Species level: reduction in *B. adolescentis* and *B. longum*
**12**	Zou et al. (2020)	48 ASD (38 male, 10 female) and 48 neurotypical children (2-7 years)	16S rRNA gene sequencing of V3-V4 regions using Illumina MiSeq platform	Phylum level: decrease in Firmicutes, Proteobacteria and Verrucomicrobia, higher Bacteroidetes/Firmicutes ratio Family level: increased *Bacteroidaceae* and *Prevotellaceae* Genus level: increased *Bacteroides*, *Prevotella*, *Lachnospiracea_incertae_sedis* and *Megamonas* and decreased *Clostridium XlVa, Eisenbergiella, Clostridium IV, Flavonifractor, Escherichia/Shigella, Haemophilus, Akkermansia*, and *Dialister* Species level: increased *Bacteroides vulgatus* and *Prevotella copri*, decreased *Bacteroides fragilis* and *Akkermansia muciniphila*
**13**	Zurita, et al. (2020)	25 ASD and 35 neurotypical children (5-12 years)	16S rRNA gene sequencing of V4 region using MiSeq Illumina platform	Genus level: increase in *Bacteroides*, *Akkermansia*, *Coprococcus* and different species of *Ruminococcus*

Most studies focused on the phylum and genus level, however, some family level analyses showed increased abundance of *Veillonellaceae*, *Pseudomonadaceae*, *Enterobacteriaceae*, *Bacteroidaceae*, *Ruminucoccaceae*, *Lachnospiraceae*, *Prevotellaceae* in individuals with ASD (Rose et al., 2018; Liu et al., 2019; Zou et al., 2020). In addition, almost 27 genera had altered abundance in ASD patients (Gondalia et al., 2012; Strati et al., 2017; Kong et al., 2019; Liu et al., 2019; Dan et al., 2020; Wang et al., 2020; Zou et al., 2020; Zurita et al., 2020) compared to neurotypical controls. Of these, some genera were highlighted in multiple clinical studies including *Bacteroides* (increased in two studies (Zou et al., 2020; Zurita et al., 2020), decreased in two studies (Niu et al., 2019; Dan et al., 2020), *Ruminococcus* increased in two studies (Wang et al., 2020; Zurita et al., 2020), decreased in a single study (Niu et al., 2019), *Bifidobacterium* increased in a sole study (Dan et al., 2020), decreased in two of the included studies (Niu et al., 2019; Wang et al., 2020), *Akkermansia* increased in one study (Zurita et al., 2020), decreased in another (Zou et al., 2020) and *Clostridium* increased in one study (Wang et al., 2020) whereas it was decreased in another report (Zou et al., 2020).

**Figure 2:**
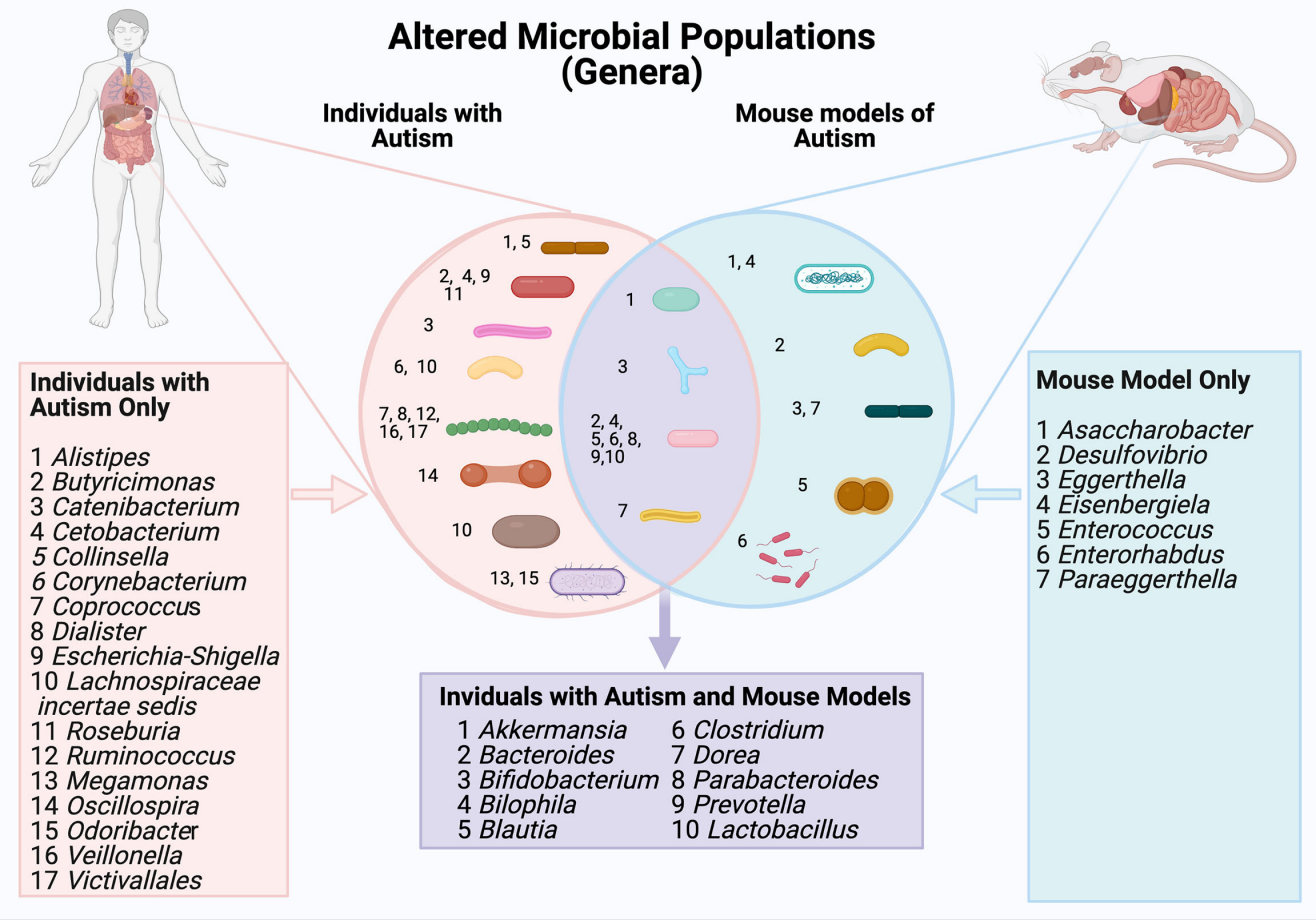
A comparison of gut microbial populations in autism: clinical and mouse models. Microbial genera are grouped by cellular morphology (i.e., spherical, rod shaped and bifurcated).

When considering morphology (**Figure 2**), the largest group of microbes that showed altered abundance were spherical (*Coprococcus, Dialister, Ruminococcus, Veillonella* and *Victivallales*) or rod shaped (*Alistipes, Butyricimonas, Catenibacterium, Cetobacterium, Collinsella, Corynebacterium, Escherichia-Shigella, Lachnospiracie incertae sedis, Roseburia, Oscillospira*).

### Predominant Properties of the GI Microbiome in Mice

Nine studies investigating the microbiome in mouse models of ASD met the selection criteria for this study. The mouse models studied in these publications were: NL3^R451C^ (Nlgn3tm1Sud/J), SHANK3 KO and 15q dup mice, and BTBR (BTBR T tf/J). Three of the included studies were conducted on models expressing ASD-associated genetic mutations (NL3^R451C^, SHANK3 KO and 15q dup mice) (Hosie et al., 2019; Sauer et al., 2019; Septyaningtrias et al., 2020), in addition to three studies of a ‘phenotype first’ model of ASD (BTBR mice) (Newell et al., 2016; Golubeva et al., 2017; Cristiano et al., 2018) as well as three studies conducted on environmental ASD mouse models that show behavioral and neuropathological symptoms relevant to autism through maternal administration of polyinosinic: polycytidylic acid (poly I:C) or valproic acid (VPA) (Lim et al., 2017), maternal immune activation (MIA) (Hsiao et al., 2013), or transplantation of microbial samples from individuals with ASD to germ-free mice (Sharon et al., 2019).

Gut microbial differences in mouse models of ASD were compared (**Table 4**). Overall, Firmicutes and Bacteroidetes were the predominant taxa at the phylum level for all ASD mouse models studied. Multiple studies report changes in the ratios of Firmicutes and Bacteroidetes in these models. A study by Lim et al. (2017) reported a small increase in Firmicutes phylum alongside a slight decrease in Bacteroidetes phylum relative to controls (Lim et al., 2017), while Hsiao et al. (2013) showed an increase in Bacteroidetes and a decrease in Firmicutes phyla (Hsiao et al., 2013). In addition, Sauer et al. (2019) reported a higher abundance of the phylum Actinobacteria and found that the phyla Deferribacteres, Chlamydiae and Tenericutes were only present in Shank3αβ KO mice when compared to controls (Sauer et al., 2019). Furthermore, offspring of germ-free mice engrafted with ASD patient microbial samples have altered expression of amplicon sequence variants within the Bacteroidetes phyla, Verrucomicrobia, α- and β-Proteobacteria phyla (Sharon et al., 2019). At the class level, Hsiao et al. (2013) reported that Clostridia and Bacteroidia are predominant in maternal immune activation (MIA) offspring (Hsiao et al., 2013) compared to untreated control mice. Furthermore, four studies on genetically modified mice (Newell et al., 2016; Golubeva et al., 2017; Hosie et al., 2019; Sauer et al., 2019) focused on mouse faecal microbial abundance at the family level found that i) *Lachnospiraceae* is more abundant in NL3^R451C^ mice, ii) a decrease in S24–7 family *Enterobacteriacea* and *Lachnospiraceae* in BTBR mice and iii) the *Bifidobacteriaceae* and *Eggerthellaceae* families are predominant in Shank3 KO mice compared to controls.

**Table 4:** Properties of the faecal microbiome in mouse models of autism.

	Publication	Sample size	Sex	Background	Mutation or environmental agent	Specimen type	Analysis method	Bacterial changes at different classification levels
**1**	Hosie et al. (2019)	5 NL3^R451C^ and 4 WT littermates	Male	B6;129‐Nlgn3tm1Sud/J	NL3^R451C^	Faecal specimen	16S rRNA gene se-quencing of V3-V4 region using Illumina MiSeq platform	Phylum level: increased Firmicutes, decreased Candidate. Family level: increased *Lachnospiraceae*
**2**	Cristiano et al. (2018)	6 BTBT and 6 B6	Male	BTBR T+tf/J (BTBR) versus C57Bl/6J (B6) (3-4 month old) mice	–	Faecal specimen	16S rRNA gene sequencing of V3-V4 region using Illumina Miseq platform	Genus level: increased *Bacteroides*, *Parabacteroides* and unknown genus *of Rikenellaceae* and decrease in unknown genera of *S24-7*, unknown genera of *Lachnospiraceae* and Clostridiales
**3**	Golubeva et al. (2017)	_	Male	C57BL/6 J (Harlan, UK) and BTBR T+ Itpr3tf/J	–	Cecum biopsy	16S rRNA gene sequencing of V3-V4 region using Illumina MiSeq platform	Phylum level: increase in Bacteroidetes and a decrease in Firmicutes Family level: decrease in *Lachnospiraceae S24-7* (Bacteroidales). Genus level: significant decrease in relative abundance of *Blautia, Desulfovibrio* and *Bifidobacterium*, and an increase in *Akkermansia*, *Bacteroides* and *Bilophila*.
**4**	Hsiao et al. (2013)	MIA offspring mice	–	Offspring of maternal immune activation (MIA) mice (Pregnant C57BL/6N mice)	Maternal immune activation	Faecal specimen	16S rRNA Gene Sequencing of V3-V5 region using454-Titanium pyrosequencer platform	Class level: increased Clostridia and Bacteroidia Order level: increased Bacteroidales Family Level: increased *Lachnospiraceae, Prevotellaceae* and *Porphyromonadaceae.*
**5**	Lim et al. (2017)	–	Male	Pregnant C57BL/6 mice	Injection of polyinosinic:polycytidylic acid (poly I:C) and valproic acid (VPA)	Faecal specimen	16S rRNA gene sequencing of V4 region using Illumina MiSeq platform	Phylum level: No sig difference in overall composition. Slight increase and decrease in Firmicutes and Bacteroidetes respectively. Family level: increase *Lachnospiraceae, Rikenellaceae, Peptostreptococcaceae*, *Enterococcaceae* and a reduction in *Prevotellaceae* Genus level: increased *Enterococcus*, *Parabacteroides*, *Dorea* reduced *Desulfovibrio, Prevotella*
**6**	Newell et al. (2016)	15 mice	Male	Juvenile BTBRT + tf/j versus C57BL/6 (B6) mice	–	Cecal and Faecal specimen	16S rRNA Gene Sequence Analysis (qPCR)	Phylum level: low Firmicutes/Bacteroidetes ratio Family level: lower *Enterobacteriaceae* Genus Level: increased *Lactobacillus* Species level increased *Akkermansia muciniphila* and *clostridium leptum*
**7**	Sauer et al. (2019)	–	–	C57BL/6 background	Shank3 KO	Faecal specimen	16S rRNA gene se-quencing of V3-V5 re-gion using Illumina MiSeq platform	Phylum level: Increased Actinobacteria and Firmicutes. Reduced levels of Proteobacteria and Verrucomicrobia. Bacteria of the phylum Deferribacteres, Chlamydiae and Tenericutes were found only in Shank3αβ KO mice. Family level: increased *Bifidobacteriaceae* and *Eggerthellaceae*, Genus level: increased *Asaccharobacter, Eggerthella, Enterorhabdus*, and* Paraeggerthella*
**8**	Septyaningtrias et al. (2020)	–	Male	C57BL/6 J background	15q dup mice	Faecal specimen	16S rRNA gene sequencing of V1-V2 region using Illumina MiSeq platform	Phylum and Genus level: No significant difference Species level: 13 OTUs predominantly belonging to the order Bacteroidales and Clostridiales were more abundant in WT mice
**9**	Sharon et al. (2019)	11 Offspring mice 11	–	Germ-free (GF) C57BL/6J	Transplanted microbiota of human ASD	Faecal specimen	16S rRNA gene sequencing of V4 region using Illumina MiSeq platform	Family level: reduced *Bacteriodetes* increased *Lachnospiraceae* Genus level: decreased *Bacteroides* and *Parabacteroides* increase in *Akkermansia* and *Sutterella*

Studies of environmental factors influencing autism-like phenotypes identified increased abundance of *Lachnospiraceae*, *Prevotellaceae* and *Porphyromonadaceae* at the family level in MIA offspring mice (Hsiao et al., 2013). Similarly, *Lachnospiraceae* also showed increased abundance in both the Poly I:C and valproic acid models together with an increased abundance of *Rikenellaceae*, *Peptostreptococcaceae*, *Enterococcaceae* (Lim et al., 2017). At the family level, an increased abundance of *Lachnospiraceae* and a decreased abundance of *Bacteriodetes* was also identified in offspring mice grafted with autistic humanized microbiome (Sharon et al., 2019).

At the genus level, a range of bacteria including *Asaccharobacter, Eggerthella, Enterorhabdus* and *Paraeggerthella* had elevated abundance in Shank3 knockout mice (Sauer et al., 2019). Similarly, in the environmental VPA model, the abundance of both *Prevotella* and *Desulfovibrio* was decreased (Lim et al., 2017). In contrast, an increased abundance of *Lactobacillus* was also reported in the BTBR mouse model of ASD compared to a reference mouse strain (Newell et al., 2016). One report also showed an increased abundance of *Akkermansia* and *Sutterella* in feces from mice that received fecal samples from individuals with ASD (Sharon et al., 2019). Incidentally, microbes modified in ASD mouse models were predominantly rod shaped (i.e., *Asaccharobacter, Desulfovibrio, Eggerthella, Eisenbergiela*, *Enterorhabdus* and *Paraeggerthella*).

### Comparison of Microbial Profiles in Autism Patients and Mouse Preclinical Models

Detailed comparisons made at multiple classification levels between ASD clinical samples and autism mouse models revealed subtle changes in the composition of the faecal microbiome. Firmicutes and Bacteroidetes remained the predominant phyla in both control and autism-relevant groups in both human and mouse studies. Differences in the Firmicutes : Bacteroidetes ratio were observed in 6 clinical studies (Strati et al., 2017; Kong et al., 2019; Liu et al., 2019; Niu et al., 2019; Dan et al., 2020; Zou et al., 2020) and in four ASD mouse studies (Newell et al., 2016; Golubeva et al., 2017; Hosie et al., 2019; Sauer et al., 2019). Interestingly, an altered abundance of Actinobacteria and Proteobacteria phylum was identified in both ASD patient studies and mouse models of ASD (Kong et al., 2019; Niu et al., 2019; Sauer et al., 2019; Dan et al., 2020; Wang et al., 2020). In both clinical studies and autism mouse models, the abundance of Actinobacteria and Proteobacteria was influenced by the presence of ASD or an ASD-associated mutation (Kong et al., 2019; Sauer et al., 2019; Dan et al., 2020; Zou et al., 2020). In addition, Clostridia and Bacteroidia classes were present at higher abundance in both ASD patients and mouse models of ASD compared to non-ASD subjects/controls (Hsiao et al., 2013; Berding and Donovan, 2020).

Gut microbial differences occurring in both ASD patients and mouse models of ASD were identified (**Table 5** and **Figure 2**). At the family level, *Peptostreptococcaceae*, *Lachnospiraceae*, *Prevotellaceae* showed altered abundance in both patient and mouse models (Hsiao et al., 2013; Golubeva et al., 2017; Lim et al., 2017; Rose et al., 2018; Hosie et al., 2019; Liu et al., 2019; Zou et al., 2020). At the genus level, the abundance of *Bilophilia*, *Clostridium*, *Dorea* and *Lactobacillus* was increased in both clinical studies and mouse models (Golubeva et al., 2017; Lim et al., 2017; Strati et al., 2017; Wang et al., 2020; Zou et al., 2020). Also at the genus level, there was altered abundance of *Akkermansia*, *Bacteroides*, *Bifidobacterium, Prevotella* and *Parabacteroides* in both clinical autistic participants and in mouse models (Golubeva et al., 2017; Cristiano et al., 2018; Niu et al., 2019; Sharon et al., 2019; Zou et al., 2020; Zurita et al., 2020). In contrast, the abundance of the genera *Blautia* was decreased in both mouse models and clinical case studies (Golubeva et al., 2017; Niu et al., 2019). Interestingly, the morphology of bacteria with altered abundance in both human and mouse model samples was overwhelmingly rod shaped (i.e.*Bacteroides, Biolphila, Blautia, Clostridium, Dorea, Parabacteroides, Prevotella, Lactobacillus*).

**Table 5:** Overlap of microbial dysbiosis profiles in individuals with ASD and preclinical models.

Bacterial Classification level	ASD clinical cases	Preclinical models of ASD	Shared characteristics of dysbiosis in ASD and preclinical models
**Phylum level**	Actinobacteria ↓/**↑** Bacteroidetes **↑**/↓ Firmicutes ↑/↓ Proteobacteria ↑/**↓** Verrucomicrobia **↓** Acidobacteria ↑/↓	Actinobacteria **↑** Bacteroidetes **↑** Firmicutes↑/↓ Proteobacteria **↓** Verrucomicrobia **↓** Candidate ↓	Actinobacteria Bacteroidetes Firmicutes Proteobacteria Verrucomicrobia **↓**
**Class level**		Bacteroidia ↑ Clostridia ↑	
**Order Level**		Clostridiales ↓	
**Family level**	*Bacteroidaceae* ↑ *Enterobacteriaceae* ↑ *Erysipelotrichacea*e↓ *Lachnospiraceae* **↑** *Peptostreptococcaceae* ↓ *Prevotelaceae* **↑** *Rikenellaceae* ↑ *Ruminucoccaceae* ↓/↑ *Streptococcaceae* ↓ *Veillonollaceae* ↑	*Bacteroides*↓ *Bifidobacteriaceae* ↑ *Clostridiaceae* *Eggerthellaceae* ↑ *Enterobacteriaceae* ↓ *Enterococcaceae* ↑ *Lachnospiraceae* ↓/**↑** *Parabacteroides* ↓ *Peptostreptococcaceae*↑ *Porphyromonadaceae* ↑ *Prevotellaceae* **↑**/↓ *Rikenellaceae* ↑ *S24-7 family (Bacteroidales)*↓	*Lachnospiraceae* *Peptostreptococcaceae* *Prevotellaceae*
**Genera level** (**Figure 1**)	*Akkermansia* **↑**/↓ *Bacteroides* **↑**/↓ *Bifidobacterium* ↑/**↓** *Bilophila* **↑** *Blautia* **↓** *Clostridium* **↑** *Dorea* **↑** *Parabacteroides* ↓ *Prevotella* ↑ *Lactobacillus* **↑** *Alistipes* ↑ *Butyricimonas -* *Catenibacterium* ↑ *Cetobacterium* ↑ *Collinsella* ↑ *Corynebacterium* ↑ *Coprococcus *↑ *Dialister* ↓ *Escherichia-Shigella* ↓ *Lachnospiraceae incertae sedis* ↑ *Roseburia* ↓ *Ruminococcus* ↑ *Megamonas* ↑ *Oscillospira* ↑ *Odoribacter* ↑ *Veillonella* ↓ *Victivallales* ↑	*Akkermansia* **↑** *Bacteroides* **↑** *Bifidobacterium* **↓** *Bilophila* **↑** *Blautia* **↓** *Clostridium* **↑** *Dorea* **↑** *Parabacteroides* ↑ *Prevotella* ↓ *Lactobacillus* **↑** *Asaccharobacter* ↑ *Desulfovibrio* ↓ *Eggerthella* ↑ *Eisenbergiela* ↑ *Enterococcus* ↑ *Enterorhabdus* ↑ *Paraeggerthella* ↑	*Akkermansia* *Bacteroides* *Bifidobacterium* *Bilophila* **↑** *Blautia* **↓** *Clostridium* **↑** *Dorea* **↑** *Parabacteroides* *Prevotella* *Lactobacillus* **↑**

Note: Arrows indicate direction of change in abundance, bold arrows indicate where direction of change is the same in both clinical ASD and mouse model.

Changes in specific components of gut microbiota profiles are observed in samples from children with ASD and across multiple mouse models of autism. Changes in the abundance of *Bacteroides, Akkermansia, Bifidobacterium, Bilophila, Blautia, Clostridium and Dorea, Parabacteroides, Prevotella and Lactobacillus* are seen in both children with ASD and mouse models (highlighted in mauve). The direction of change in abundance levels in each of these clinical samples/models are specified in **Table 5**.

## Discussion

We conducted a systematic review of microbial diversity in ASD patients and in mouse models of ASD. Twenty-two studies were included (13 articles describing dysbiosis in clinical cases of ASD and 9 articles focused on microbial shifts in ASD mouse models) which further validate mouse models with respect to observations from clinical cohorts.

### Clinical Microbial Dysbiosis in ASD

Microbial dysbiosis profiles identified from clinical ASD cases have been associated with intestinal inflammation and GI symptoms such as constipation and diarrhea, which are commonly observed in individuals with ASD (Adams et al., 2011; Gorrindo et al., 2012; Rose et al., 2018; Pagani et al., 2019). Clinical reports highlighted an increased abundance of numerous bacterial genera in stool samples from individuals with ASD, including pathogenic microbes linked to GI inflammation and severe symptoms for ASD patients such as *Clostridium, Blautia, Dialister, Escherichia-Shigella, Prevotella* and *Streptococcus*. Others, such as microbes belonging to the *Akkermansia* genus of mucin-degrading bacterium, were increased in prevalence in ASD individuals, and are known to affect the structure of the gastrointestinal tract through metabolic activities and correlate with changes in gut permeability (Wang et al., 2011; Dao et al., 2016). In contrast, the abundance of members of the *Bacteroides* genus (microbes providing beneficial carbohydrate fermentation associated with the production of the neurotransmitter gamma aminobutyric acid (GABA) in the gut (Strandwitz et al., 2019) was increased in ASD clinical samples.

The current study concurs with previous assessments of the literature demonstrating a variance in microbial diversity of individuals with ASD. Similar to the systematic review by Iglesias-Vázquez et al., (2020) that focused on the phylum and genera level, we identified changes in the predominance of Firmicutes and Bacteroidetes, as well as altered abundance of Proteobacteria, Actinobacteria and Verrucomicrobia in stool samples of ASD patients. As per our findings, these authors also showed an increase in *Bacteroides, Parabacteroides* and *Clostridium*. In contrast, Iglesias-Vázquez et al., 2020 reported a decreased abundance of *Coprococcus* and *Bifidobacterium* in stool samples from ASD patients, whereas we identified studies with increases in their abundance (Dan et al., 2020; Zurita et al., 2020). Additionally, a review by Srikantha & Mohajeri (2019) reported altered microbial diversity in ASD patients with increases in the Firmicutes to Bacteroidetes ratio, and an increase in abundance of *Clostridium* genera, similar to observations in the current review.

Of particular interest, we noted an increased abundance of *Clostridium* in several clinical studies. In individuals with autism, an increased abundance of *Clostridium*, a gram-positive anaerobic bacterium, is associated with increased GI inflammation in the colon (Song et al., 2004; Finegold et al., 2010; Luna et al., 2017). *Clostridium* spp. have multiple virulence factors, which include extracellular toxins (alpha, beta, epsilon and iota toxin) and hydrolytic enzymes (Popoff and Poulain, 2010). A higher abundance of Clostridium isolated from the faecal specimens of individuals with ASD compared to that of control subjects has been reported in multiple studies (Hsiao, 2014; Argou-Cardozo and Zeidán-Chuliá, 2018). Relevant to ASD populations, Emanuele et al. (2010) identified higher levels of enterotoxin in serum samples from ASD patients than controls and therefore proposed that these enterotoxins significantly contribute to severe GI symptoms in autistic individuals (Emanuele et al., 2010).

In addition, we report altered abundance (increased in one study and decreased in two) of the probiotic *Bifidobacterium. Bifidobacteria* regulate immune activity *via* fermentation of complex polysaccharides in the gastrointestinal tract and as such are believed to exert positive health benefits on the host (Menard et al., 2005; Popoff and Poulain, 2010; Dinan et al., 2013; Delcarua et al., 2016). Similarly, both increases and decreases in abundance were reported in *Ruminococcus*, an important bacterium for the digestion of starch. Relevant to this review, abundance of *Ruminococcus* has been proposed as an indicator for irritable bowel syndrome (Malinen et al., 2010).

Overall, analyses of stool samples from participants diagnosed with ASD revealed an imbalance of gut microbial communities including bacteria previously reported to alter gut function. Although precise biological mechanisms are unknown, microbial alterations may contribute to GI symptoms and behavioral changes in ASD *via* gut-brain axis dysfunction.

### Preclinical Microbial Dysbiosis in Mouse Models of ASD

Firmicutes and Bacteroidetes phyla comprise the predominant murine microbial community, making up more than 70% of the microbial profile. Importantly, this finding was consistent across model construct (i.e., mice expressing autism-associated genetic mutations or subjected to environmental factors associated with ASD-relevant phenotypes in mice such as maternal immune disorders or valproic acid exposure). Nevertheless, we observed subtle differences in predominant bacterial phyla in each of the ASD mice models, which likely reflects differences in genetic mutations and environmental factors. In a study by Lim and colleagues, mice exposed to polyinosinic: polycytidylic acid (poly I:C) or valproic acid (VPA) showed a subtle increase in the abundance of Firmicutes and a decrease in abundance of Bacteroidetes (Lim et al., 2017). In contrast, BTBR mice showed an increased abundance of bacteria within the Bacteroidetes phylum and a decrease in bacteria belonging to the Firmicutes phylum (Cristiano et al., 2018). In BTBR mice, a decrease in *Bifidobacterium* and *Blautia* abundance has been shown to reduce bile-metabolism due to impaired tryptophan metabolism in the gut (Golubeva et al., 2017). This observation further indicates that microbial dysbiosis may lead to dysregulation of host-microbial interactions and contribute to pathophysiological outcomes. In addition, a higher abundance of the phylum Actinobacteria alongside a significantly lower abundance of the phyla Proteobacteria and Verrucomicrobia were reported in faecal samples from Shank3αβ KO mice (Sauer et al., 2019). In the same preclinical model, microbes within the phyla Deferribacteres, Chlamydiae and Tenericutes were present exclusively in Shank3αβ KO mice and not controls (Sauer et al., 2019). Collectively, these findings show that the microbial community is altered in a variety of mouse models of ASD.

In the majority of ASD mouse models assessed, an increased abundance of potentially pathogenic microbes (i.e., correlating with severe gastrointestinal symptoms in patients such as *Clostridium*) and a decreased abundance of probiotic microbes (which play an important role in immunity as well as in maintaining physiological homeostasis of the gut ecosystem) such as *Bifidobacterium* and *Blautia* was observed.

### A Comparison of Clinical and Preclinical Microbial Profiles in ASD

When comparing across species, most human and mouse microbiome studies reported an altered Bacteroidetes : Firmicutes ratio at the phylum level. At the family level, *Lachnospiraceae* and *Prevotellaceae* showed increased abundance in clinical studies but showed variation in the mice studies with both increased and decreased changes in abundance. Conversely, *Peptostreptococcaceae* had decreased abundance in clinical studies and increased abundance in mice studies. At the genus level, an increased abundance of microbes such as *Clostridium, Bacteroides, Bilophila, Dorea* and *Parabacteroides* were observed in both mouse models of autism and autistic individuals. However, the abundance of *Bifidobacterium* and *Blautia* genera fluctuated across ASD patient studies but were decreased in several mouse models of autism. This variation in findings could be due to species, genetic and/or environmental factors that directly affect the microbial ecosystem within the gastrointestinal tract. It is therefore important to study microbial diversity in mouse models where external/influencing factors can be more readily controlled and to characterize responses to specific gene mutations or environmental insults.

## Conclusions

Here we highlight common cross-species alterations of gut microbial profiles in clinical cases of ASD and mouse models of autism. We demonstrate an increase in abundance of genus level microbes such as *Clostridium, Lactobacillus, Dorea* and *Bilophilia* and a decrease of *Blautia* in both the clinical setting and in mouse models of autism. Given the presence of these changes across both clinical and preclinical studies, these microbial profiles could potentially contribute to GI symptoms observed in individuals with ASD. This systematic review provides further support for preclinical models as tools to identify potential therapeutic targets for treating gastrointestinal disorders in ASD.

## Author Contributions

SH, AF, EH-Y conceived the study. MA conducted the literature searches with support from SH and EH-Y. AS, JW and AF contributed to the interpretation of microbial results. All authors drafted the manuscript. All authors contributed to the article and approved the submitted version.

## Funding

This research was funded by an Australian Research Council Future Fellowship to EH-Y (FT160100126) and a National Health and Medical Research Council Ideas grant to EH-Y and AF (APP2003848).

## Conflict of Interest

The authors declare that the research was conducted in the absence of any commercial or financial relationships that could be construed as a potential conflict of interest.

## Publisher’s Note

All claims expressed in this article are solely those of the authors and do not necessarily represent those of their affiliated organizations, or those of the publisher, the editors and the reviewers. Any product that may be evaluated in this article, or claim that may be made by its manufacturer, is not guaranteed or endorsed by the publisher.
